# Supplementary material for: Comparison of the Immune Responses to COVID-19 Vaccines in Bangladeshi Population
Source: Vaccines (Basel). 2022 Sep 8;10(9):1498. doi: 10.3390/vaccines10091498 (PMC9504987; doi:10.3390/vaccines10091498)

Supplementary Materials

Communication

Comparison of the Immune Responses to COVID-19 Vaccines in Bangladeshi Population

Protim Sarker [1], Evana Akhtar [1], Rakib Ullah Kuddusi [1], Mohammed Mamun Alam [1], Md. Ahsanul Haq [1], Md. Biplob Hosen [1], Bikash Chandra Chanda [2], Farjana Haque [1], Muntasir Alam [1], Abdur Razzaque [3], Mustafizur Rahman [1], Faruque Ahmed [4], Md. Golam Kibria [4], Mohammed Zahirul Islam [5], Shehlina Ahmed [6] and Rubhana Raqib [1,*]

[1] Infectious Diseases Division, International Centre for Diarrhoeal Disease Research (icddr,b), Dhaka 1212, Bangladesh
[2] Laboratory Sciences and Services Division, International Centre for Diarrhoeal Disease Research (icddr,b), Dhaka 1212, Bangladesh
[3] Health Systems and Population Studies Division, International Centre for Diarrhoeal Disease Research (icddr,b), Dhaka 1212, Bangladesh
[4] Sheikh Russel Gastroliver Institute & Hospital, Dhaka 1212, Bangladesh
[5] Embassy of Sweden in Dhaka, Dhaka 1212, Bangladesh
[6] Foreign Commonwealth & Development Office (Bangladesh), Dhaka 1212, Bangladesh
* Correspondence: rubhana@icddrb.org

Supplementary Figure S1: Flow chart showing specimen collection, and use of specimens for different analysis.

Supplementary Table S1: Cell surface markers used in flowcytometric analysis

Supplementary Figure S2: Gating strategy for T and B cell panels

Supplementary Figure S1: Flow chart showing specimen collection, and use of specimens for different analysis.

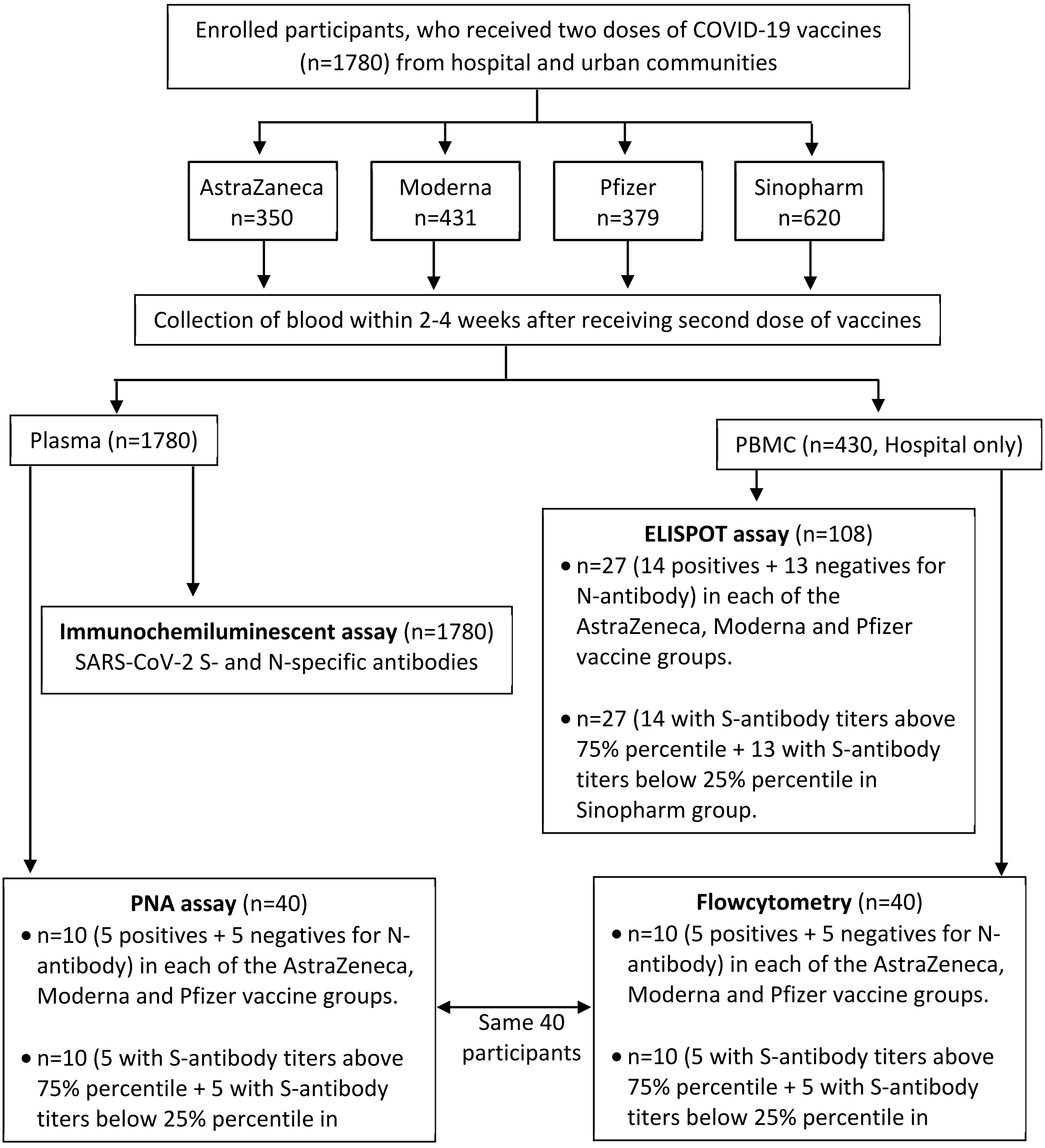

Supplementary Table S1. Cell surface markers used in flowcytometric analysis of T and B cell subtypes

**Cell types**	**Abbreviation**	**Cell surface markers**
**T cell subtypes**		
Pan T cells	CD3^{+} T cells	CD3^{++}
T-helper cells	CD4^{+} T cells	CD3^{+}CD4^{+}
T- cytotoxic cells	CD8^{+} T cells	CD3^{+}CD8^{+}
Regulatory T cells	T$_{reg}$ cells	CD3^{+}CD4^{+}CD25^{++}CD127^{-}
T helper naive cells	CD4^{+} T$_{N}$ cells	CD3^{+}CD4^{+}CD45RA^{+}CD45RO^{-}CCR7^{+}
T helper effector cells	CD4^{+} T$_{EFF}$ cells	CD3^{+}CD4^{+}CD45RA^{+}CD45RO^{-}CCR7^{-}
T helper effector memory cells	CD4^{+} T$_{EM}$ cells	CD3^{+}CD4^{+}CD45RA^{-}CD45RO^{+}CCR7^{-}
T helper central memory cells	CD4^{+} T$_{CM}$ cells	CD3^{+}CD4^{+}CD45RA^{-}CD45RO^{+}CCR7^{+}
Cytotoxic naive T cells	CD8^{+} T$_{N}$ cells	CD3^{+}CD8^{+}CD45RA^{+}CD45RO^{-}CCR7^{+}
Cytotoxic T effector cells	CD8^{+} T$_{EFF}$ cells	CD3^{+}CD8^{+}CD45RA^{+}CD45RO^{-}CCR7^{-}
Cytotoxic T effector memory cells	CD8^{+} T$_{EM}$ cells	CD3^{+}CD8^{+}CD45RA^{-}CD45RO^{+}CCR7^{-}
Cytotoxic T central memory cells	CD8^{+} T$_{CM}$ cells	CD3^{+}CD8^{+}CD45RA^{-}CD45RO^{+}CCR7^{+}
**B cell subtypes**		
Lymphocyte		CD45^{++}
Dead cell		Annexin B^{++}
Live cells		Annexin B^{--}
B cells		CD19^{++} CD3^{-}
Early B cell		CD19^{+}CD38^{++}
Regulatory B cell	B$_{reg}$ cells	CD19^{+}CD24^{++}CD27^{+}
Memory B cell		CD19^{+}CD27^{++}
Plasmablast		CD19^{+}CD20^{+}CD38^{+}CD27^{+}

*All antibodies were purchased from BD Biosciences (San Jose, USA)

Supplementary Figure S2: Gating strategy for T and B cell panels

A. T cell panel

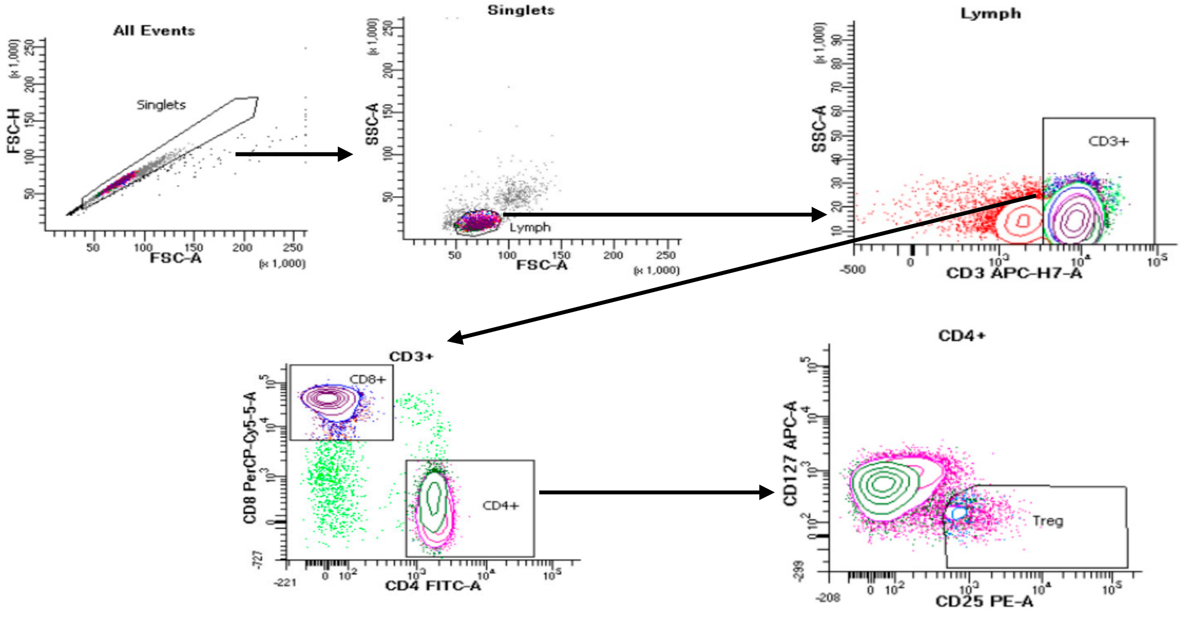

B. B cell panel

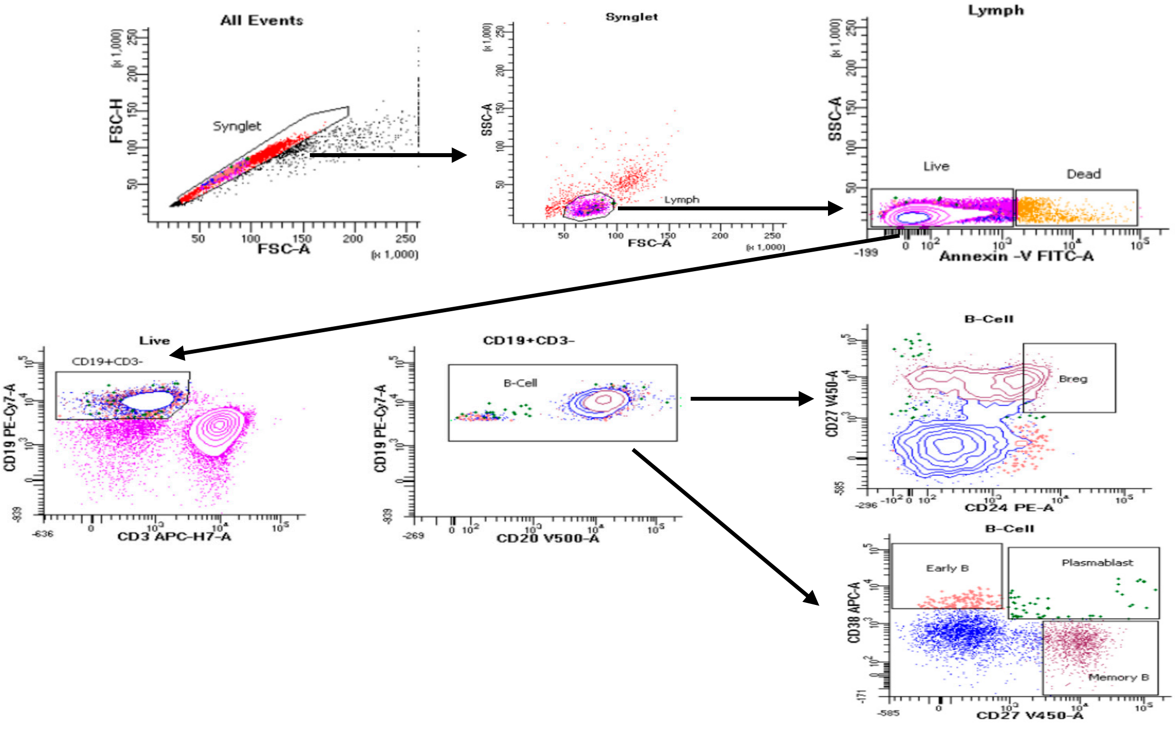

Supplement: Supplementary file 1 [file vaccines-10-01498-s001.zip › vaccines-1863389-supplementary.pdf]
